# Effect of physical training on parathyroid hormone and bone turnover marker profile in relation to vitamin D supplementation in soccer players

**DOI:** 10.5114/biolsport.2022.109956

**Published:** 2021-11-10

**Authors:** Michał Brzeziański, Monika Migdalska-Sęk, Michał Stuss, Zbigniew Jastrzębski, Łukasz Radzimiński, Ewa Brzeziańska-Lasota, Ewa Sewerynek

**Affiliations:** 1 Medical University of Lodz, Department of Endocrine Disorders and Bone Metabolism, 90-752 Lodz, Poland; 2 Medical University of Lodz, University Laboratory of Three-Dimensional Anthropometry, 92-213 Lodz, Poland; 3 Medical University of Lodz, Department of Biomedicine and Genetics, 92-213 Lodz, Poland; 4 Gdansk University of Physical Education and Sport, Department of Physiology and Biochemistry, 80-336 Gdansk, Poland

**Keywords:** Soccer players, Physical training, PTH, Bone turnover markers, Vitamin D3 supplementation, Osteocalcin, CTx

## Abstract

The aim of the study was to assess the impact of vitamin D supplementation and regular physical activity on 25-hydroxyvitamin D [25(OH)D], parathyroid hormone (PTH) and bone turnover marker concentrations in healthy male athletes. Twenty-five youth soccer players were divided into groups: non-supplemented (GN) and supplemented (GS) with a vitamin D dose of 20 000 IU twice a week for 8 weeks. The study was conducted during an 8-week preseason period, from mid-January to mid-March. At baseline (T1) and at the end of this period (T2), the serum concentrations of 25(OH)D, (PTH), osteocalcin (OC) and β-isomerized C-terminal telopeptide of type I collagen (β-CTx) were measured. At T2, 25(OH)D increased by 70% in GS (*p* = 0.004) and by 6% in GN (*p* > 0.05). Significant differences between GS and GN groups were observed throughout the study in the group-by-time interaction and changes of 25(OH)D (*p* = 0.002; *η*^2^*p* = 0.36) and OC (*p* = 0.008; *η*^2^*p* = 0.26). Increased OC (ES = 0.74; moderate) and β-CTx (ES = 1.31, large) in GN athletes who had an optimal baseline vitamin D level (GO) were observed. In GN, at T2, β-CTx positively correlated with PTH and OC (*p* = 0.007 and *p* = 0.002). In GS, β-CTx positively correlated with OC at both time points (T1, *p* = 0.027 and T2, *p* = 0.037). A negative correlation between 25(OH)D and PTH was observed at T2 (*p* = 0.018). The obtained results suggest that the 20 000 IU vitamin D3 dose applied twice a week for 8 weeks is effective for vitamin D compensation and sufficient to maintain the correct PTH concentration, as revealed by changes in the bone marker concentrations. In conclusion, the results suggest that the applied vitamin D supplementation dose in athletes leads to intensive bone remodelling and has protective effects on bone under intensive physical effort.

## INTRODUCTION

It was documented that the active form of vitamin D, calcitriol (1,25-dihydroxyvitamin D), has pleiotropic effects in many tissues and organs, including bones and skeletal muscles and regulates approximately 1000 different genes [1, 2]. Under the influence of solar radiation, 7-dehydrocholesterol is transformed into previtamin D, and next it is hydrolysed in the liver to 25-hydroxyvitamin D [25[OH D]. It is the most important form of vitamin D used in evaluation of vitamin D status. Calcitriol controls the body’s phosphate and calcium homeostasis and is responsible for the maturation of osteocytes. Vitamin D deficiency leads to significant impairment of skeletal mineralization [3, 4]. The importance of vitamin D in determining human health has been widely studied in the general population [2, 4], but similar studies focused on athletes are rare. However, it is believed that an adequate level of vitamin D is also very important in the group of athletes, affecting the proper functioning of many body systems, including the musculoskeletal system.

Today, vitamin D has become a key ingredient in the athlete’s diet. Many athletes use vitamin D supplements as part of their daily diet. The most important beneficial effects of vitamin D supplementation in athletes are: optimization of muscle function and remodelling [5, 6], maintaining bone metabolism [7], and reducing the risk of injury [8, 9]. Vitamin D supplementation has been shown to affect muscle performance, kinetics and efficiency during exercise adaptation [5, 6, 10]. It has previously been confirmed that professional athletes have insufficient serum vitamin D levels [11, 12]. In professional basketball, approximately 32% of athletes are vitamin D deficient, and 47% are insufficient [13]. Among the National Football League players it was found that 26% were deficient in vitamin D, and 42% to 80% of athletes were vitamin D insufficient [14]. Similarly, in many different populations of athletes, deficiencies or insufficiencies have been found in the majority of diagnosed soccer players, hockey players, dancers, taekwondo fighters, jockeys, elite wheelchair athletes, handball players, track and field athletes, weightlifters, swimmers and volleyball players [11, 15, 16]. Intensive physical effort together with vitamin D deficiency intensifies catabolic processes in the muscle tissue [5, 6]. This process results in impaired cross-bridge formation and muscle weakness [17], which in turn may impair athletes’ performance [5, 6]. It has also been suggested that vitamin D deficiency may be associated with a disturbance of bone mineralization in response to intensive physical activity [18–20].

A practical method of early detection of bone cell responses to physical effort is the measurement of the bone turnover markers (BTM). Their serum concentrations reflect the activity of remodelling processes occurring in the whole skeleton over time [21]. One of the most important proteins in non-collagenous bone tissue, synthesized by osteoblasts under the influence of an active vitamin D metabolite, is osteocalcin (OC). It is a marker of bone formation, especially mineralization, characterized by a high affinity for calcium ions in hydroxyapatite crystals, and participates in the binding of calcium salts and their deposition in the bone matrix [22, 23]. The OC concentration reflects the intensity of osteogenesis and is positively correlated with the histomorphometric parameters of the bone formation process [24]. In turn, the β-isomerized C-terminal telopeptide of type I collagen alpha chain (β-isomerized C-terminal telopeptide of type I collagen; β-CTx) released during osteoclastic bone resorption occurs in all tissues in which type I collagen forms pyridinoline cross-linking. Although it is not specific for bone tissue, it is assumed that the vast majority comes from bone resorption and is a good predictor of fractures [25].

It has been confirmed that in response to repeated mechanical loading, bone turnover increases, due to both bone formation and remodelling that involves osteocytes [20, 23, 24]. It is also speculated that physically active people have a higher bone turnover compared to non-athletic people [23, 26, 27]. In one of the studies it was proved that the levels of OC in the serum were respectively 14% and even 60% higher in football players and decathlon competitors than in the control group [7, 26]. The main influence on bone remodelling and physical adaptation is the force of gravity (e.g. climbing stairs, running, walking) and secondly, the strength of the muscles (e.g. weightlifting, swimming). In addition, exercise intensity and duration may alter bone turnover [28].

While it is known that long-term moderate intensity exercise has a beneficial effect on bone resorption [29], in the case of high-intensity exercises in competitive sports, the situation is different. Some researchers observed only a transient increase in bone cell activity, as assessed by measuring the concentration of osteocalcin and CTx, in response to intense and prolonged exercise [30]. However, no significant changes in the concentrations of osteocalcin and type I collagen metabolites were observed during a short-term maximum physical effort [31]. On the other hand, the importance of exercise for maintaining normal bone mass and structure was confirmed by the fact that the 6–8-week rest period after the end of the football season causes inhibition of bone formation and a parallel induction of bone resorption [32, 33], which negatively affects bones.

It has already been shown that an alteration of BTM is more related to training loads than to the changes in vitamin D levels, which did not significantly correlate with the levels of BTM [34, 35]. In addition, in one of the studies it was documented that a significant decrease in the level of 25(OH)D after the winter period did not cause any significant changes in the levels of BTM [P1NP (procollagen I aminoterminal propeptide), OC, β-CTx, OC/β-CTx] [35] while in the second one there were higher levels of P1NP (another bone formation marker) in winter compared to summer, although no changes in CTx were observed [36].

It is documented that vitamin D stimulates bone formation and increases the level of parathyroid hormone (PTH), which stimulates the activity of osteoclasts in the bone [28, 37]. Its main function is to maintain the physiological concentration of calcium ions in extracellular fluids. This hormone could reflect bone remodelling, but its involvement in more specific actions, referred to as catabolic and anabolic, is also well known [21, 38]. At the cellular level, PTH promotes bone resorption, mainly by acting on the receptor activator of nuclear factor κ-B (RANK) and its ligand (RANKL), a part of the RANKL/RANK/osteoprotegerin pathway, leading to an increase in osteoclast formation and activity. PTH-induced bone formation is mediated, inter alia, by a decrease in *SOST*/sclerostin expression in osteocytes [20, 39]. Intermittent injection of PTH has been suggested to exert an anabolic effect at three stages of bone formation: (1) stimulating preosteoblast proliferation; (2) promoting preosteoblast and osteoblast differentiation; and (3) inhibiting osteoblast apoptosis [40].

Literature data from studies focusing on the relationship between PTH and exercise show that physical effort is an important modifier of PTH concentration depending on the intensity, duration, recovery and type of exercise [21]. Interestingly, it seems that metabolic factors modulating PTH secretion are: pH, metabolic acidosis and catecholamines [26, 41]. Moreover, the regulation of PTH is also influenced by age, gender, initial bone mineral content and physical fitness [21]. Several studies have shown that exercises of gradually increasing intensity until exhaustion [42] and continuous (2 exercises lasting 21 minutes each at 70 and 85% VO_2max_, respectively) or intermittent (2 exercises of 21 minutes each at 70 and 85% VO_2max_, respectively, separated by 40 minutes recovery) sub-maximal exercise [38] all increase PTH levels. A marked increase in PTH levels was also noted in young men during prolonged (> 50 minutes) and intense (15% above the ventilation threshold) [30] or very long (5 hours) exercise and low intensity (50% VO_2max_) [43], which suggests that minimal intensity and duration are needed to induce a change of PTH concentration. However, it has been reported in elderly population studies that PTH levels increased after maximum incremental exercise testing and that this increase may have an anabolic effect on bone turnover [18]. On the other hand, a long-term (50 minutes) low-intensity exercise (15% below the ventilation threshold) [30] or short-term (30 seconds) maximum exercise [31] does not appear to have an effect on PTH secretion.

In the light of the cited studies, little attention is devoted to the analysis of the relationship between vitamin D, PTH levels, and bone turnover marker (BTM) serum concentrations in athletes in response to exercises. According to published data from the above-mentioned studies, the results concerning the influence of vitamin D supplementation on the BTM and PTH values are inconclusive. It should be emphasized that these changes depend on many physiological factors and the exercise load during physical exertion is essential. Considering the complexity of the feedback mechanisms in maintaining the proper calcium-phosphate homeostasis, it would be important not only to recognize the direct effect of the defined optimal dose of vitamin D3 supplementation on the BTM concentrations (bone formation and resorption), but also to know how and whether BTM and PTH will change, under the influence of physical effort and/or vitamin D supplementation. So far, studies on the optimal dose of vitamin D3 supplementation in Polish athletes and its influence on the BTM profile are also still inconsistent [7, 27, 35]. Therefore, in our research, we hypothesised that vitamin D3 supplementation and regular physical activity have a synergistic effect, preventing micro-fractures and the loss of bone mass or even causing its increase during physical exertion in young athletes. In the mechanism of increasing the absorption of calcium and phosphorus in the intestines and their reabsorption in kidneys, vitamin D3 supplementation significantly stimulates osteoblasts and inhibits PTH secretion. To evaluate this hypothesis, we assessed the concentrations of 25(OH]D, PTH and bone turnover markers (OC, β-CTx, OC/β-CTx) after vitamin D3 supplementation (20 000 IU twice a week for 8 weeks) in young athletes practising football training.

## MATERIALS AND METHODS

### Experimental approach

The study was approved by the Medical University of Lodz Ethics Committee (RNN/283/18/KE, 18 September 2018), and the investigation was carried out following the rules of the Declaration of Helsinki of 1975. All players and their parents or legal guardians were provided with detailed information about the study procedures and gave their written consent. The training plan of athletes was developed at the Arka Gdynia football club in Gdynia, Poland. Laboratory tests were carried out at the Department of Endocrine Disorders and Bone Metabolism and at the Department of Biomedicine and Genetics of the Medical University of Lodz.

### Characteristics of the study group

Twenty-five soccer players from an under-19 team participated in the study. All athletes had a valid athlete’s health card. Selected biological features of athletes and selected training characteristics are shown in Table 1. The soccer players enrolled in the study were in the period which during the experiment included 7 training sessions (Monday–Friday) and one friendly game (Saturday) a week. The study was conducted during an 8-week preseason period, from mid-January to mid-March Each workout began with a warm-up. Previously, the athletes implemented the standard soccer training plan developed by the team’s training staff. Training loads carried out at the first stage of preparations (4 weeks) included low and medium intensity exercises (with a predominance) of aerobic and mixed (aerobic-anaerobic) metabolism. At the second stage of preparation (4 weeks), the training loads were aerobic-anaerobic and anaerobic. At this stage, high-intensity exercises based on the interval method (e.g. small-sided games) and repetition (e.g. shaping the motion speed) were dominant. Training was monitored on the basis of training diaries and training plans arranged by trainers. Only players who participated in at least 85% of training sessions and matches were considered in the final analysis.

**TABLE 1:** Selected biological characteristics of soccer players included in the study and selected training characteristics.

Parameter	Total group (TG)
Age [years]	17.5 ± 0.70
Body height [cm]	height 178 ± 0.70
Body weight [kg]	weight 68.05 ± 9.18
Training period [weeks]	8 weeks in the preparation period (winter, from mid-January to mid-March)
Training unit [min]	each training session lasted 90 minutes, 5 training units and a control (sparring) match each week, In addition, the players participated in 2 units of physical education lessons at school (90 minutes) with a focus on soccer practice

The study inclusion criteria were: male gender and possession of an up-to-date athlete’s health card, i.e., they were free of any pathology or clinical conditions. They were not taking medications two weeks before the data collection and vitamin supplementation for 1 month before the study. Additionally, athletes had to follow a sports diet suggested by a club nutritionist and demonstrate 80% attendance during the experiment.

The criteria for exclusion from the experiment were female gender, use of stimulants, e.g. caffeine, drugs, smoking, taking medications, chronic diseases, sports injuries during the experiment, low physical capacity resulting from training absenteeism or an injury 1 month before the experiment, and lack of athlete’s or their parents’ or legal guardians’ written consent. The competitors were informed about the purpose of the research project.

### Vitamin D3 supplementation and analysis

The examined athletes (total group; TG) were randomly divided into two groups: with D supplementation (GS) and without vitamin D supplementation (GN). No significant differences in 25(OH)D were detected between the groups.

– Group I (n = 12) – subjected to training and supplemented with vitamin D3 (cholecalciferol in a dose of 20 000 IU – Decristol, Sun-Farm Sp. z o.o.) which was taken in a dose 20 000 IU vitamin D3 twice a week for 8 weeks; supplementation was carried out under the supervision of a sports dietitian, during the preparation of the annual training cycle (from January to March).
– Group II (n = 13) – subjected to training only, without vitamin D3 supplementation.

The dispensation procedure was double-blind. Supplementation and testing were carried out in late winter, when vitamin D synthesis was likely to be minimal. During the study, the subjects were asked several times if they had taken the vitamins as instructed. All the subjects reported that they had followed the instructions. It was confirmed by the measurements of 25(OH)D before and 8 weeks after the supplementation. During the experiment, 70% of the players fed themselves in the school boarding house and 30% at home. The diet of the players was low in vitamin D. The estimated consumption during the experiment was about 150 UI/day for competitors eating at home and about 200 UI/day for competitors eating in the boarding school. This value was estimated on the basis of a calculator of vitamin D content in food products, during seven days in the middle of the experiment, based on the list of products consumed by the athletes.

Additionally, based on the results of the 25(OH)D blood test, GN and GS groups were divided depending on the baseline of 25(OH)D concentration into subgroups: with a suboptimal 25(OH) D concentration (< 30 ng/ml, GSO; n = 9 and n = 8, respectively) and with the optimal 25(OH)D concentration (≥ 30 ng/ml, GO; n = 4) [44].

### Blood and serum collection

The collection of biological material was conducted by nurses according to the reliable protocols of collection, transport, and storage of biological material. Five millilitres of whole arm vein blood was collected from each athlete in both groups (GN and GS), at baseline (time point T1) and at the end of the study, i.e., after 8 weeks of the training cycle and/or cholecalciferol supplementation (time point T2).

The blood was collected in sterile tubes, marked with an identification number, without anticoagulant (on the so-called clot). The blood samples were allowed at room temperature (approximately 30–45 minutes) to form a clot. After centrifugation at 2400 rpm for 10 minutes at room temperature, the serum (supernatant) was carefully separated from the clot into Eppendorf tubes. The obtained biological material was frozen at -20°C.

### Evaluation of serum 25-hydroxyvitamin D concentration

The method of evaluation of serum 25-hydroxyvitamin D [25(OH)D] concentration was based on the commercial kit Elecsys Vitamin D Total II (Roche Diagnostics GmbH). The ECLIA automated electrochemiluminescence method was performed on the COBAS analyser (the cobas e411 System). Total 25(OH)D is the combination of 25[OH]D2 plus 25[OH]D3. The reaction and evaluation of 25(OH)D concentration (ng/ml) were carried out at 20°C. Fifteen millilitres of the analysed serum sample was incubated with the reagents for the initial sample preparation: PT1 – containing dithiothreitol, and with reagents PT2 – containing sodium hydroxide. In this step, under the influence of PT2, vitamin D (25-OH) is released from VDBP (vitamin D binding protein). Then, after incubation of the sample with reagent R1, which contains ruthenium-labelled VDBP, a complex of vitamin D (25-OH) – ruthenium-labelled VDBP – is formed. Subsequent incubation with streptavidin-coated microparticles (reagent M) and biotinylated 25-hydroxyvitamin D (reagent R2) produces a complex consisting of ruthenium bound vitamin D binding proteins and biotinylated vitamin D (25-OH). The formed “sandwich-type” complex, based on the affinity of biotin and streptavidin, binds to the solid phase (magnetic bonding of particles to the surface of the platinum electrode). Unbound substances are removed with ProCell buffer. During the chemiluminescence reaction, the concentration of the sample was measured against a standard curve (Roche/Hitachi COBAS e 411; produced in 2018), using a photomultiplier. Reference values for 25(OH)D in serum were: 30–70 ng/ml normal value; 20–30 ng/ml slight deficiency; 10–20 ng/ml moderate deficiency; < 10 ng/ml severe deficiency. The assay sensitivity was 4.01 ng/ml. The inter-assay coefficients of variation (CV) range was between 2.20% and 10.70% across a working range of 3.00–70.00 ng/ml for four serum standards in 84 samples.

### Evaluation of serum parathyroid hormone (PTH) and bone turnover marker concentrations (beta form of cross-linked C-terminal telopeptide of type I collagen (β-CTx), osteocalcin (OC))

The principle of determination of PTH, β-CTx and OC in blood serum was based on commercial reagent kits from Roche Diagnostics GmbH, respectively, for the analyte under study: Elecsys PTH (1–84), Elecsys β-CrossLaps/serum and Elecsys N-MID Osteocalcin. The automated concentration measurement method is an immunoenzymatic competitive method, in which the total application time was 18 minutes and the whole reaction and measurement was carried out at 20°C. The analysed blood serum samples (50 µL each for PTH and CTx and 20 µL for OC) were incubated with R1 reagent, which contains biotinylated monoclonal antibodies specific respectively for the analysed analytes: N-terminal fragments of parathormone, type I collagen degradation fragments containing octapeptides in the ßisomeric form irrespective of the nature of the cross-links between these octapeptides. The samples were then subjected to a second incubation. The samples were then subjected to a second incubation with streptavidin-coated microparticles (reagent M) and monoclonal antibodies appropriate for the analytes tested: the C-terminal fragment of PTH, anti-β-CrossLaps, anti-N-MID osteocalcin labelled with ruthenium complex (reagent R2).

The formed sandwich-type complex, based on the affinity of biotin and streptavidin, binds to the solid phase. After automatic mixing, the reaction mixture is transferred to the measuring chamber. By means of a magnet, the microparticles are attracted to the surface of a platinum electrode. Unbound substances are removed with Pro-Cell buffer. The voltage applied to the electrode induces a chemiluminescence reaction and photon emission, measured with a photo-multiplier. The concentration of the test analyte was read from the analyser calibration curve based on a 2-point calibration and the standard curve contained in the barcode of the reagent.

Reference values for PTH in serum: 14.90–56.90 pg/ml; assay sensitivity 6.0 pg/ml. The inter-assay coefficients of variation (CV) range was 3.0–6.5% across a working range of 1.2–5000.0 pg/ml for three serum standards in 60 samples. Reference values for β-CTx in serum of 14–18-year-old men: 0.54–2.035 ng/ml; assay sensitivity 0.07 ng/ml. The inter-assay coefficients of variation (CV) range was 1.6–4.7% across a working range of 0.01–6.0 ng/ml for three serum standards in 60 samples. Reference values for OC in serum < 30-year-old men: 24–70 ng/ml; the inter-assay coefficients of variation (CV) range was 1.8–6.5% for three serum standards in 60 samples.

### Statistical data analysis

Statistical analysis was performed using Statistica version 13.1 software (TIBCO Software Inc. 2017, Cracow, Poland). Results were presented as mean ± standard deviation (SD). For all study parameters in the study groups the Shapiro-Wilk test was used to test for normality (n < 50) and the Levene test was conducted to check the uniformity of the variables analysed. The t-test was applied for variables with a normal distribution. When data distribution was not compatible with a normal distribution, statistical differences were assessed using nonparametric tests (Mann-Whitney U test and Wilcoxon pairwise test). Additionally, a two-factor analysis of variance (ANOVA) was performed for repeated measurements. This analysis was used to assess both inter-group and intra-group effects. Moreover, partial eta squared (η^2^p) was calculated to determine the magnitude of the effect. The η^2^p was classified as small (≥ 0.01), medium (≥ 0.06) and large (≥ 0.14). Moreover, Cohen’s D was calculated to check statistical differences in the subgroups. The effect size (ES) was classified as trivial (< 0.2), small (> 0.2–0.6), moderate (> 0.6–1.2), large (> 1.2–2.0) and very large (> 2.0–4.0) [45]. Lastly, in order to evaluate the relationship for study variables, Spear-man’s rank correlation coefficient was used. The significance level for all analyses was set at p < 0.05.

## RESULTS

### Changes in 25(OH)D, PTH and bone turnover marker (BTM) concentrations in the studied groups after supplementation with vitamin D3 and physical training

Table 2 shows the mean ± SD of 25(OH)D, BTM and PTH at two time points (T1, T2) in each group of athletes. A statistically significant difference in the group-by-time interaction of 25(OH)D between the two groups (GN and GS) was found (*p* = 0.002; η^2^*p* = 0.36). Furthermore, a significant group effect (*p* = 0.006; η^2^*p* = 0.28) and time interaction (*p* = 0.0003; *η*^2^*p* = 0.44) for 25(OH)D were found. At point T2 (i.e., after 8 weeks of training and/or cholecalciferol supplementation), 25(OH)D levels increased for both GS and GN, by 70% and 6%, respectively. A significant increase in 25(OH)D levels at T2 vs. T1 for the supplemented group (GS) was confirmed (*p* = 0.001).

**TABLE 2:** 25(OH)D, PTH and BTM concentrations in the groups of athletes: total group (TG), supplemented with vitamin D3 group (GS) and non-supplemented group (GN) at baseline (T1) and after 8 weeks (T2).

Group	TG		GN		GS		Interactions	*p*	*η* ^2^ *p*
Time point	T1	T2	T1	T2	T1	T2			
25(OH)D[ng/ml]	26.68 ± 10.01	36.88 ± 15.74*	25.54 ± 9.52	27.15 ± 12.06	27.92 ± 10.79	47.42 ± 12.21*^&^	group	0.006	0.28
							time	0.0003	0.44
							group × time	0.002	0.36

PTH [pg/ml]	28.04 ± 8.52	25.40 ± 8.38	25.21 ± 7.83	22.45 ± 7.39	31.11 ± 8.48	28.59 ± 8.50	group	0.046	0.16
							time	0.091	0.12
							group × time	0.938	0.00

Osteocalcin[ng/ml]	56.32 ± 23.19	56.16 ± 19.23	45.46 ± 13.24	49.77 ± 15.50*	68.08 ± 26.30^&^	59.64 ± 18.21	group	0.028	0.19
							time	0.832	0.01
							group × time	0.008	0.26

β-CTx[ng/ml]	1.08 ± 0.32	1.14 ± 0.31	0.95 ± 0.20	1.08 ± 0.15*	1.23 ± 0.35^&^	1.22 ± 0.40	group	0.065	0.14
							time	0.187	0.07
							group × time	0.106	0.11

OC/β-CTx[ng/ml]	52.84 ± 16.67	49.63 ± 13.70	50.21 ± 18.12	45.73 ± 10.67	55.70 ± 12.68	53.86 ± 15.75	group	0.196	0.07
							time	0.276	0.05
							group × time	0.646	0.01

Data are presented as mean value ± standard deviation.

* : Significant differences (*p* < 0.05) over time (T1 vs. T2) within the same group.

& : Significant differences (*p* < 0.05) between groups (GN vs. GS) in the same study point. TG – total group, GN – non-supplemented group, GS – supplemented group, 25(OH)D – serum concentrations of twenty-five hydroxyvitamin D, PTH – parathyroid hormone, β-CTx – β-isomerized C-terminal telopeptide of type I collagen, OC – osteocalcin.

PTH concentration at T2 decreased for both GS and GN by 9% and 12%, respectively. There were no significant differences in the group-by-time interaction of PTH (*p* > 0.05). However, analysis of variance indicated significant group effect for this variable (*p* = 0.046; η^2^*p* = 0.16).

Analysis of the results of bone turnover marker (BTM) concentrations showed a significant group effect (*p* = 0.028; *η*^2^*p* = 0.19) and group-by-time interaction (*p* = 0.008; *η*^2^*p* = 0.26) for OC. There were no significant differences in the group-by-time interaction β-CTx (*p* > 0.05; *η*^2^*p* = 0.11). In the non-supplemented group (GN), statistically significantly higher levels of both OC and β-CTx at T2 compared to T1 were observed (*p* = 0.005 and *p* = 0.037, respectively). OC concentrations increased by 9%, while β-CTx concentrations increased by 14%. In contrast, the concentrations of both BTMs were slightly decreased at T2 compared to T1 in the supplemented group. The differences in the concentrations of the tested markers were not statistically significant (*p* > 0.05). At baseline (T1), OC as well as β-CTx concentrations were significantly higher in GS vs. GN (*p* = 0.011 and *p* = 0.021, respectively).

Values of bone turnover rate index (OC/β-CTx) were decreased at T2 compared to T1 (*p* > 0.05) by 10% in GN and 3% in GS. The analysis of variance did not show significant differences in the group-by-time interaction for this variable (*p* > 0.05; *η*^2^*p* = 0.01).

### Comparison of PTH and BTM concentration before and after training (T1 vs. T2) in the studied groups: GS (supplemented) and GN (non-supplemented) depending on vitamin D3 supplementation

The supplemented (GS) and non-supplemented (GN) groups were divided according to baseline vitamin D3 concentration by subgroups: with suboptimal 25(OH)D concentration (< 30 ng/ml; GSO) and with optimal 25(OH)D concentration (≥ 30 ng/ml; GO). The results are presented in Table 3.

**TABLE 3:** PTH and BTM concentrations in the group of athletes with suboptimal 25(OH)D concentration (< 30 ng/ml; GSO) and in the group with optimal 25(OH)D concentration (≥ 30 ng/ml; GO) at baseline (T1) and after 8 weeks (T2).

Group	GN						GS					
Subgroup	GSO		ES	GO		ES	GSO		ES	GO		ES
Time-point	T1	T2		T1	T2		T1	T2		T1	T2	
PTH [pg/ml]	26.66 ± 8.97	23.48 ± 8.46	0.27	21.95 ± 3.20	20.14 ± 4.18	0.51	27.22 ± 6.08	27.64 ± 8.46	0.06	38.88 ± 7.53	30.49 ± 9.53	0.92

Osteocalcin [ng/ml]	46.56 ± 15.21	50.44 ± 18.66	0.24	43.00 ± 8.52	48.25 ± 5.25	0.74	61.63 ± 22.35	60.25 ± 20.38	0.07	81.00 ± 32.21	68.75 ± 24.39	0.85

β-CTx [ng/ml]	1.01 ± 0.16	1.08 ± 0.18	0.36	0.80 ± 0.23	1.08 ± 0.07	1.31	1.13 ± 0.35	1.18 ± 0.48	0.12	1.43 ± 0.31	1.29 ± 0.19	0.57

OC/β-CTx	45.99 ± 11.75	46.20 ± 12.59	0.02	59.71 ± 27.77	44.69 ± 5.53	0.74	55.65 ± 13.41	54.43 ± 17.46	0.08	55.80 ± 13.03	52.71 ± 14.00	0.24

Note: Data are presented as mean value ± standard deviation, GN – non-supplemented group, GS – supplemented group, GSO – group with sub-optimal level of 25(OH)D, GO – group with optimal level of 25(OH)D, ES – effect size, PTH – parathyroid hormone, β-CTx – β-isomerized C-terminal telopeptide of type I collagen, OC – osteocalcin

In the GO subgroup of GS a decrease of PTH by 28% at T2 vs. T1 was observed (ES = 0.92; moderate). In GN, both in GSO and GO PTH levels were decreased at T2 compared to T1 (ES = 0.27 and 0.51, respectively; small).

Analysing the results of OC concentrations in GN, an increase of 8% was observed in GSO (ES = 0.24; small) and 12% in GO (ES = 0.74; moderate) at T2. In contrast, a decrease in OC concentrations was observed in GS in both subgroups, with a moderate ES for GO and trivial ES for GSO. In GN at T2, the concentrations of β-CTx increased by 35% in GO (ES = 1.31, large), while in GS a decrease of β-CTx by 10% in the GO subgroup was observed (ES = 0.57; small). The values of bone turnover rate index (OC/β-CTx) decreased or increased at T2 vs. T1 depending on the subgroup (see Table 3).

### Correlations between concentrations of 25(OH)D, PTH and BTM in the group supplemented with vitamin D3 (GS) and the non-supplemented group (GN) at baseline (T1) and after 8 weeks (T2)

Table 4 shows data regarding statistically significant correlations (p < 0.05; R = 0.06–0.79, moderate-high) between the studied parameters in the two groups throughout the study. In the non-supplemented group (GN), positive correlations were found between β-CTx and PTH and β-CTx and OC concentrations at T2. In contrast, in the supplemented group (GS), β-CTx concentration positively correlated with OC concentration at both time points (T1 and T2). Additionally, time point T1 showed statistically significant associations between 25(OH)D and β-CTx and 25(OH)D and PTH concentrations, whereas time point T2 showed a statistically significant negative correlation between 25(OH)D and PTH.

**TABLE 4:** Correlations between concentration of 25(OH)D, PTH, OC and β-CTx in the group supplemented with vitamin D3 (GS) and the non-supplemented group (GN) at baseline (T1) and after 8 weeks (T2).

Group	Time point	Parameter	Parameter	R	*p*
GN	T2	PTH	β-CTx	0.707	0.007
	T2	Osteocalcin	β-CTx	0.773	0.002

GS	T1	25(OH)D	PTH	0.643	0.024
	T1	25(OH)D	β-CTx	0.717	0.009
	T2	25(OH)D	PTH	–0.667	0.018
	T1	Osteocalcin	β-CTx	0.634	0.027
	T2	Osteocalcin	β-CTx	0.606	0.037

## DISCUSSION

The purpose of this study was to assess the influence of vitamin D3 supplementation and regular physical activity on 25(OH)D, PTH and bone turnover marker (OC, β-CTx, OC/β-CTx) concentrations in young athletes participating in football training. Vitamin D is known to control calcium absorption from the gastrointestinal tract, and even mild vitamin D deficiency – compensated by an increase in serum PTH – results in enhanced bone turnover, which in turn may increase the risk of microinjuries or fractures [20].

Studies, focused mainly on animal material, suggest that a proper supply of vitamin D, as indicated by 25(OH)D concentration, can optimize myocyte function, as well as muscle contraction strength and power. Moreover, it accelerates muscle recovery, which will translate into better athletic performance. In turn, strong and well-mineralized bones will better protect the athlete from potential fractures [5, 6, 28]. Unfortunately, despite the evidence for the beneficial effects of vitamin D, current research is inconclusive regarding optimal intake, especially in athletes. As in the general population [44], it is believed that the optimal serum vitamin D level in athletes is 30–50 ng/ml and should be maintained throughout the whole year. Maintaining such a level prevents rickets and osteomalacia, falls and bone fractures and contributes to improved sports performance [19, 28, 46]. According to some authors, only levels of 25(OH)D > 40 ng/ml ensure optimal functioning of the musculoskeletal system and most effectively prevent fractures. On the other hand, increasing the 25(OH)D level above 50 ng/ml does not seem to bring any measurable health benefits [19, 46] and a further increase in its supplementation may have negative effects, resulting, for example, from the risk of overdose, although the current guidelines encourage obtaining higher concentrations, but < 100 ng/ml [47].

Considering the recommendations for the general population [44], a large proportion of athletes are vitamin D deficient or insufficient, regardless of the sport discipline [11, 12, 15]. These observations also apply to Polish soccer players, among whom about 38–60% were found to be vitamin D deficient [7, 10]. It was not different in judo competitors, 80% of whom had baseline 25(OH)D concentration < 30 ng/ml [16]. In the soccer players included in our study, the baseline mean 25(OH)D concentration was 26.68 ng/ml, which can be described as mild deficiency or a suboptimal concentration [44].

The objectives of the athlete health strategy include the elimination of abnormalities in bone metabolism, among others in bone mineralization, to which vitamin D deficiency undoubtedly leads. Therefore, at the beginning of our study, we hypothesized that baseline vitamin D levels in athletes (before starting vitamin D supplementation) would influence the BTM profile at the end of the training cycle in the supplemented group. In our experiment, after 8 weeks of training and supplementation of 20 000 IU of cholecalciferol twice weekly (GS), 25(OH)D concentrations significantly increased from 27.92 to 47.42 ng/ml, i.e. by 70%. Participants in the supplemented group achieved 25(OH)D concentrations > 30 ng/ml, which clearly demonstrates the effectiveness of the regimen used in compensating for the deficiency of the aforementioned vitamin. Most of the cholecalciferol supplementation strategies presented in the literature for the athletic population are effective in compensating for deficiency or maintaining adequate vitamin D concentrations in the body [6, 10, 48].

Limited studies have focused on the relationship between vitamin D status and BTM in the Polish athlete population [7, 27, 35]. In our study, the bone turnover rate index (OC/ß-CTx) decreased by 3–10% after the training cycle, depending on the studied group of athletes. Despite a more pronounced decrease in the OC/ß-CTx index after the training period, which is demonstrated by moderate ES, while maintaining optimal 25(OH)D levels (GO group) we did not observe any significant differences in the bone turnover rate. This may indicate maintaining a balance between bone resorption and formation processes during intensive exercises. In other studies conducted on a group of Polish football players, no significant relationships between the concentrations of 25(OH)D and BTM were found [7, 35].

In contrast to the authors of these studies, we suggest that the increase in BTM in GN was related not only to training but also to vitamin D supply. Our conclusion is supported by the observed increase in OC and β-CTX concentrations in subgroups distinguished according to baseline vitamin D supply (GSO and GO) subjected only to physical burden. Furthermore, in athletes with optimal 25(OH)D vitamin D levels (GO), training increased BTM levels, which is demonstrated by a moderate to large ES. We interpret the obtained results, especially in the context of vitamin D supplementation, as favourable, because they probably indicate inhibition of over-intensive bone remodelling under the influence of excessive physical exertion, which may be accompanied by bone micro-damage, leading to fatigue fractures. Literature data indicate a higher risk of fractures in American football players with vitamin D deficiency [14]. Furthermore, a protective effect of vitamin D3 supplementation on stress fractures was observed during the training of female Navy recruits [49]. On the other hand, NBA players who experienced previous stress fractures were found to have significantly higher 25(OH)D concentrations, which can be interpreted as a secondary preventive effect in the form of increased supply of the aforementioned vitamin [50].

Apart from proper supplementation with components having a beneficial influence on the locomotor system, also physical activity of adequate intensity, frequency and duration of the load or impact can maximize the bone formation process [51]. It is known that accelerated bone turnover, with the predominance of bone formation, affects primarily the processes of structural modelling of bones and their internal remodelling, i.e. changes in their microarchitecture leading to an increase in their strength [20, 23]. The assessment of bone metabolism seems to be important in the light of the continuous adaptation of bones to the loads exerted on them, especially when higher values of the OC/ß-CTx ratio are obtained in the group of professional football players than in the group of physically inactive men, and higher 25(OH)D levels positively correlate with body weight, total body water, lean mass and muscle mass [7]. In our study, the observed group-time interaction and, additionally, the group effect suggest that GS and GN were significantly different with respect to OC concentration, but in the non-supplemented group, i.e. subjected only to intensive physical training, there was a significant increase in both studied markers, i.e. OC and β-CTx.

Moreover, we found a strong positive correlation between the studied BTM, which indicates intensification of bone remodelling processes due to high load on the skeletal system and is in accordance with the hypothesis of Maïmoun et al. [26], who associate dynamic changes occurring in the skeleton manifested by high levels of both resorption and formation markers with the necessity to repair bone microinjuries. In contrast, in the group receiving vitamin D3, we observed only a slight, not statistically significant decreasing trend in concentrations of both markers after the training cycle. However, the concentrations of both studied markers, i.e. OC and β-CTx, were significantly higher at T1 in the group undergoing later supplementation, which may have had a significant impact on the results. On the other hand, some authors have concluded that the decrease in bone turnover in response to physical exertion may be related to either decreased bone formation or decreased resorption or increased renal clearance [23].

The literature data on the BTM levels (OC, β-CTx, OC/β-CTx) in the context of high physical activity are inconsistent and their concentrations depend on the type of exertion. Bones of athletes training in team games are subjected to high or at least moderate loads, which is reflected in higher concentrations of bone formation markers, e.g. BALP (bone alkaline phosphatase) [52], OC, P1NP, but also the resorption marker CTx [53]. This is in agreement with our results in GN, where OC levels increased by 9% and β-CTx by 14%. Other studies show increased bone formation, but with a concomitant decrease of resorption markers, which suggests that the intensification of bone remodelling may be strongly influenced by resistance and weight-bearing exercise, which theoretically should lead to intensive bone remodelling and increased bone strength. In one study, after 6 months of regular activity consisting of a combination of aerobics (60%), resistance exercise (weightlifting) (30%), and flexibility-enhancing exercise (stretching) (10%), trained subjects had a 9.8% increase in OC and a 16% decrease in CTx [29]. In our study, the football players’ training was mixed and included concentric-eccentric exercises with a slight resistance component, which in part explains the slightly different BTM profile.

In contrast, other changes in BTM can be expected in individuals with a low level of physical fitness. In a group of inactive young women subjected to an eight-week resistance and mixed training programme, increased bone formation was observed without significant changes in resorption markers [51]. Interestingly, discontinuation of training may result in a significant increase in the resorption marker CTx, and a significant decrease in the bone formation marker OC [32, 33], i.e. changes characteristic for adynamic bone tissue involution.

It appears that the sex of young players training in the same sports discipline (soccer) is an independent factor modulating the BTM profile in the context of physical burden and vitamin D status. In a cross-sectional study of a population of adolescent Swedish female soccer players, with a mean 25(OH)D concentration of 20 ng/ml, the OC concentration was 59.4 ng/ml and ß-CTx was 1.075 ng/ml [54]. A similar BTM profile was presented by the young football players enrolled in our study. However, in contrast to our results, the authors did not detect any significant correlations between the aforementioned parameters. The whole training period in the population of all our athletes did not cause any change in mean OC concentration, which remained under 56 ng/ml, while ß-CTx concentration slightly increased to 1.14 ng/ml, but this change was not significant. Janik et al. [24] in a prophylactic programme of vitamin D3-supplemented postmenopausal women subjected to interval training on a bicycle ergometer, in contrast to our results, found an increase of OC concentrations and insignificant changes of β-CTx levels, while obtaining optimal values of 25(OH)D concentrations after exercise. These data confirm that the alterations of bone turnover are dependent not only on the type of training, but also the age of the trainee may be crucial in the response of bone cells to exercise.

PTH, which can exert both catabolic and anabolic effects on bone [21, 38, 39], also plays a key role in the regulation of bone metabolism. In our study, neither the supplementation nor the intense training significantly affected PTH concentrations. The entire training period led to an insignificant decrease in PTH concentrations compared to baseline. Only one study has confirmed a decrease in PTH concentrations dependent on vitamin D supplementation, but with a concomitant supply of calcium through the training period. Interestingly, in contrast to our study, the authors noted a significant increase in PTH in the placebo group, thus highlighting the benefits of combined Ca and vitamin D supplementation for maintaining bone health during periods of increased bone turnover, such as intense training [20]. A typical metabolic stimulator of PTH secretion is calcium and vitamin D deficiency, which can lead to secondary hyperparathyroidism [55]. However, in the case of a slight severity or absence of the above-mentioned disorders, often the changes in PTH may be short-term and transient with the end of the effort [56]. In our study, PTH concentrations were decreased by 9% in GS and 12% in GN and remained within normal limits after training. With the hormone secretion remaining physiological, it is difficult to make any strong conclusions about the metabolic effect of PTH after physical effort.

It is important to emphasize the presence of an inverse correlation between 25(OH)D and PTH concentrations at T2 in the supplemented group and a decrease of 28% in PTH concentration after the training cycle in the subgroup with optimal vitamin D supply (GO). It is well known that an adequate supply of vitamin D prevents the occurrence of secondary hyperparathyroidism, which has a negative impact on bone metabolism and may lead to bone loss and impair bone mineralization [55].

This result also supports the beneficial effect of vitamin D supplementation used in our study on bone metabolism in these athletes, although the concentration of 25(OH)D sufficient to maintain PTH levels in a range that will not cause bone mass loss is still unclear. Cross-sectional studies confirm that suppression of PTH levels reaches a plateau above 25(OH)D concentrations of approximately 30 ng/ml, although this relationship is not maintained in every subject. Furthermore, 25(OH)D concentrations above 30 ng/ml do not appear to be associated with an additional change in PTH secretion, and a 25(OH)D threshold of 20 ng/ml is sufficient for PTH suppression in individuals with normal renal function [37].

Opinions are also divided regarding the effect of exercise on PTH concentrations [30, 31, 38]. Some authors suggest the existence of a certain “bone stimulation threshold” including both duration and intensity of exercise, which, if exceeded, could cause a decrease in ionized calcium concentration and stimulate PTH secretion. The decrease in calcium levels, especially ionized calcium, during exercise is thought to be the main factor responsible for the increase in PTH, but catecholamine secretion and acidosis accompanying intense exercise are likely to play an important role as well [30]. An increase in PTH probably also causes sensitization of bone cells to mechanical stimuli [57]. Bouassida et al. [38] observed that ionized calcium concentration decreases during continuous exercise, while PTH increases and remains elevated 24 hours after cessation of activity. In addition, the recovery period influences the normalization of calcium and PTH concentrations, and according to the authors, the described relationships may be evidence of increased anabolic processes occurring in the bones. In addition, Maimoun et al. [30] reported that exercise in cyclists with an intensity of 15% above the ventilatory threshold (VT) causes a significant increase in PTH levels after the last minute of the test and a further increase during the 15-minute recovery. These observations highlight the important role of physical activity in the processes of osteogenesis.

## STUDY LIMITATIONS

We aware that our study has significant limitations, mainly due to the small size of the group. Finally, only 63% of players were included in the analysis, because in soccer during the season the composition of the team changes due to injuries or transfers. Additionally, despite the fact that our population included young and healthy athletes, there is a possibility that individuals may have suffered from asymptomatic diseases significantly affecting vitamin D absorption and bone metabolism. In addition, a limitation of the study is the inability to assess bone mineral density (densitometry), which might have helped us to identify athletes at high risk of fractures.

## CONCLUSIONS

The 8-week supplementation regimen of 20 000 IU of cholecalciferol twice a week was effective in compensating for vitamin D deficiency. However, we interpreted the significant reduction in concentrations of resorption (ß-CTx) and bone formation (OC) markers in the supplemented group compared to their significant increase in the group subjected only to intensive training as a beneficial effect. In our opinion, an adequate supply of vitamin D3 seems to prevent negative changes in bone metabolism or negative bone balance caused by intense physical activity. It seems reasonable to monitor the levels of 25(OH)D in soccer players, especially after the winter period, when exposure to sunlight is significantly limited. We assume that that adequate vitamin D supply and regular physical activity have a synergistic effect, preventing loss of bone mass, and in some cases have a pro-anabolic effect, increasing bone strength. However, more extensive research is required to verify our hypothesis and identify mechanisms responsible for that effect.
